# *Stenotrophomonas maltophilia* Phenotypic and Genotypic Diversity during a 10-year Colonization in the Lungs of a Cystic Fibrosis Patient

**DOI:** 10.3389/fmicb.2016.01551

**Published:** 2016-09-30

**Authors:** Arianna Pompilio, Valentina Crocetta, Dipankar Ghosh, Malabika Chakrabarti, Giovanni Gherardi, Luca Agostino Vitali, Ersilia Fiscarelli, Giovanni Di Bonaventura

**Affiliations:** ^1^Department of Medical, Oral, and Biotechnological Sciences, “G. d'Annunzio” University of Chieti-PescaraChieti, Italy; ^2^Center of Excellence on Aging and Translational Medicine (CeSI-MeT), “G. d'Annunzio” University of Chieti-PescaraChieti, Italy; ^3^Special Center for Molecular Medicine, Jawaharlal Nehru UniversityNew Delhi, India; ^4^Department of Medicine, Campus Bio-Medico UniversityRome, Italy; ^5^Microbiology Unit, School of Pharmacy, University of CamerinoCamerino, Italy; ^6^Children's Hospital and Research Institute “Bambino Gesù”Rome, Italy

**Keywords:** cystic fibrosis, lung infections, *Stenotrophomonas maltophilia*, chronic infection, biofilm, virulence, antibiotic-resistance

## Abstract

The present study was carried out to understand the adaptive strategies developed by *Stenotrophomonas maltophilia* for chronic colonization of the cystic fibrosis (CF) lung. For this purpose, 13 temporally isolated strains from a single CF patient chronically infected over a 10-year period were systematically characterized for growth rate, biofilm formation, motility, mutation frequencies, antibiotic resistance, and pathogenicity. Pulsed-field gel electrophoresis (PFGE) showed over time the presence of two distinct groups, each consisting of two different pulsotypes. The pattern of evolution followed by *S. maltophilia* was dependent on pulsotype considered, with strains belonging to pulsotype 1.1 resulting to be the most adapted, being significantly changed in all traits considered. Generally, *S. maltophilia* adaptation to CF lung leads to increased growth rate and antibiotic resistance, whereas both *in vivo* and *in vitro* pathogenicity as well as biofilm formation were decreased. Overall, our results show for the first time that *S. maltophilia* can successfully adapt to a highly stressful environment such as CF lung by paying a “biological cost,” as suggested by the presence of relevant genotypic and phenotypic heterogeneity within bacterial population. *S. maltophilia* populations are, therefore, significantly complex and dynamic being able to fluctuate rapidly under changing selective pressures.

## Introduction

*Stenotrophomonas maltophilia* is one of the most common emerging multi-drug resistant pathogens found in the lungs of people with cystic fibrosis (CF) where its prevalence is increasing (Amin and Waters, 2014; Green and Jones, 2015; Salsgiver et al., 2016). Nevertheless, it is unclear whether *S. maltophilia* simply colonizes the lungs of people with CF without adverse effect or causes true infection leading to pulmonary inflammation and clinical deterioration. Clinical studies reported conflicting results on the correlation between the presence of this microorganism and lung damage (Karpati et al., 1994; Goss et al., 2002). It has recently been shown that chronic infection with *S. maltophilia* in people with CF is an independent risk factor for pulmonary exacerbation requiring hospitalization and antibiotics and was associated with a systemic immune response to *S. maltophilia* (Waters et al., 2011).

In a series of studies, we found evidence highly suggestive of the pathogenic role of *S. maltophilia* in CF patients. This microorganism can grow as biofilm not only on abiotic surfaces (Di Bonaventura et al., 2004, 2007a,b; Pompilio et al., 2008) but also on CF-derived epithelial monolayer (Pompilio et al., 2010), probably because of a selective adaptation to CF airways (Pompilio et al., 2011). Furthermore, in a murine model of acute respiratory infection we observed that *S. maltophilia* significantly contributes to the inflammatory process resulting in compromised respiratory function and death (Di Bonaventura et al., 2010).

In the diseased CF lung, pathogens are exposed to a complex range of selection pressures including host physiological factors, oxygen tension, immune responses, therapeutic antimicrobials, and competing microorganisms. Together, these are thought to drive genetic and phenotypic diversity in the pathogen over time. Consequently, various airway-specific adaptations are postulated to favor persistence and lead to host-tolerant clonal lineages that are less cytotoxic, better at evading the immune system, more resistant to antimicrobials and less metabolically active than their ancestral strains (Hill et al., 2005; Bragonzi et al., 2009; Behrends et al., 2013). These studies have been largely focused on *Pseudomonas aeruginosa* (Hogardt and Heesemann, 2010; Hauser et al., 2011). In comparison, the adaptation of *S. maltophilia* in the CF lung has been investigated rarely (Vidigal et al., 2014), and is largely unknown.

In order to understand the adaptive strategies developed by *S. maltophilia* for chronic colonization of the CF lung, we systematically characterized 12 temporally isolated strains from a single CF patient over a 10-year period. We studied their relative growth rate, biofilm formation, motility, mutation frequencies, antibiotic resistance spectrum, virulence, and pathogenicity. We report for the first time that chronic *S. maltophilia* displays unusual adaptive plasticity by modulating its virulence and pathogenicity, yet exacerbating antibiotic resistance and other factors that augment its fitness in the CF lungs.

## Materials and methods

### Bacterial strains and growth conditions

Thirteen *S. maltophilia* isolates, collected during 11 year-period (2004–2014) from sputum of a CF patient (ethically coded “ZC”) at the CF Unit of “Bambino Gesù” Children's Hospital and Research Institute of Rome, were investigated in this study. One strain per year was considered, except than for 2012 and 2013 when two strains were obtained during the same year. The patient was selected owning to clinically defined chronic infection with *S. maltophilia*, which mandates at least 50% of samples must be positive in the preceding 12 months (Pressler et al., 2011). *S. maltophilia* was co-cultured with *P. aeruginosa* in 2010, 2011, and 2014 only. Each strain was identified by the Vitek automated system (bioMérieux Italia SpA; Florence, Italy), then stored at −80°C until use, when it was grown at 37°C in Trypticase Soy broth (TSB; Oxoid SpA; Garbagnate M.se, Milan, Italy) or Mueller-Hinton agar (MHA; Oxoid) plates. *S. maltophilia* ATCC13637 reference strain, and *S. maltophilia* Sm111, knock-out for *fliI-*gene (Pompilio et al., 2010), were used as controls in mutation frequency and motility assays, respectively.

### Bacterial genotyping

The epidemiological relatedness of the strains was studied by pulsed-field gel electrophoresis (PFGE), as previously described (Pompilio et al., 2011). Agarose-embedded DNA was digested with the restriction enzyme *XbaI*, and then separated with 6 V/cm for 20 h at 12°C, with pulse times 5–35 s and an included angle of 120°. PFGE profiles were analyzed by visual inspection and isolates were considered as belonging to the same PFGE cluster if they differed by ≤ 3 bands (Gherardi et al., 2015). Isolates with indistinguishable PFGE profiles belonged to the same pulsotype.

### Growth rate

Overnight cultures in TSB were corrected with fresh TSB to an OD_550_ of 1.00, corresponding to about 1–5 × 10^9^ CFU/ml. This suspension was diluted 1:100 in fresh TSB, then 200 μl were dispensed in each well of a microtiter plate (Kartell SpA; Noviglio, Milan, Italy), and incubated at 37°C, under static conditions, in a microplate reader (Sinergy H1 Multi-Mode Reader; BioTek Instruments, Inc., Winooski, VT, USA). OD_550_ readings were taken every 30 min for 24 h. Considering the exponential growth phase selected on a graph of ln OD_550_ vs. time (*t*), mean generation time (MGT) was calculated as follows: MGT = ln2/μ, where μ (growth rate) = (lnOD_*t*_ − lnOD_*t*0_)/*t*.

### Biofilm formation

Biofilm formation was assayed as described by Pompilio et al. (2008). Two-hundred microliters of the 1:100 diluted inoculum (prepared as described in “Growth Rate”) were dispensed to each well of a flat bottom 96-well polystyrene tissue culture-treated plate (Falcon BD; Milan, Italy), and incubated in static culture at 37°C for 24 h. Samples were washed twice with PBS (pH 7.3; Sigma-Aldrich Co., Milan, Italy), then crystal violet-stained biomass was quantified by measuring the optical density at 492 nm (OD_492_). Biofilm biomass was normalized on the growth rate by calculating the “Specific Biofilm Formation” (SBF) index as follows: SBF = biofilm biomass (OD_492_)/growth rate (μ).

### Motility

Swimming, swarming, and subsurface twitching assays were performed as described by Rashid and Kornberg (2000), with modification. Swimming and swarming were assessed by surface inoculating a single colony onto swimming agar (10 g/l tryptone, 5 g/l NaCl, 3 g/l agar) or into swarming (8 g/l nutrient broth, 5 g/l dextrose, and 5 g/l agar) agar. After incubation at 37°C for 24 h, the growth zone was measured in millimeters. Twitching was measured by inoculating a single colony to the bottom of Petri dish containing 1% TSB solidified with 1% agar. Twitch zones were stained with crystal violet after 72 h of incubation at 37°C, and measured in millimeters.

### Mutation frequencies

Mutation frequency of each strain was assessed according to Oliver et al. (2000), with modification. For each sample, three tubes containing 20 ml of Mueller-Hinton broth (Oxoid) were inoculated with one independent colony, obtained from overnight-growth on MHA plate, and incubated overnight with agitation (130 rpm). Samples were centrifuged (4500 rpm, 10 min, 4°C) and pellets resuspended in 1 ml of Mueller-Hinton broth. Ten-fold dilution of each sample was seeded onto MHA plates (controls) and onto MHA added with rifampin (Sigma-Aldrich) 250 μg/ml. Colony counts were performed after 24 h of incubation of the MHA plates and after 48 h of incubation of the MHA-rifampin plates. Mutation frequency was calculated as the number of rifampin-resistant colonies in proportion to the total viable count. Strains were classified into four categories based on mutation frequency (*f*) (Turrientes et al., 2010): hypo-mutators (*f* ≤ 8 × 10^−9^), normo-mutators (8 × 10^−9^ < *f* < 4 × 10^−8^), weak-mutators (4 × 10^−8^ ≤ *f* < 4 × 10^−7^), and strong-mutators (*f* ≥ 4 × 10^−7^).

### Virulence assays

The virulence potential of *S. maltophilia* strains was evaluated both *in vivo* in *Galleria mellonella* larvae, and *in vitro* on human A549 alveolar basal epithelial cells. (i) *Galleria mellonella* infection assays were performed as described by Betts et al. (2014), with minor modifications. Overnight cultures of *S. maltophilia* grown in TSB were washed and resuspended in PBS. Twenty larvae were inoculated with each *S. maltophilia* strain at doses of 10^3^, 10^4^, 10^5^, and 10^6^ CFU/larva, or PBS only (controls). Ten microliters of the bacterial suspension or PBS were injected directly into the hemocoel of the wax moth via the right proleg using 10-μL Hamilton syringe (Hamilton Co., Nevada, USA). Larvae were incubated in the dark at 37°C and checked daily for survival until 96 h. Larvae were considered dead if they failed to respond to touch. A “pathogenicity score” was assigned to each strain, considering both time and dose needed to achieve LD_50_. The higher the score, the higher the virulence.

(ii) *S. maltophilia* co-culture infection assays on human respiratory epithelial cells were performed according to Karaba et al. (2013), with minor modifications. Human A549 alveolar basal epithelial cells (ATCC CCL-185) were seeded at 10^5^ cells/ml in 24-mm diameter cell culture polyester inserts in 6-well Transwell™ plates (Corning; USA). Monolayers were grown overnight (37°C, 5% CO_2_) in DMEM (high glucose) with 1% penicillin, streptomycin, amphotericin (HiMedia; Mumbai, India), and supplemented with 10% fetal bovine serum (Invitrogen, USA). Each *S. maltophilia* strain, grown in TSB medium, was added to each well at Multiplicity-Of-Infection (MOI) of 500 on the apical surface of the monolayer insert. The co-culture sets were incubated for 24 h at 37°C in 5% CO_2_, gently washed to remove suspended bacteria and dead epithelial cells, and then subjected to Live/Dead^TM^ assay (ThermoFisher Scientific; Rodano, Milan, Italy). Cell death, cell rounding, and loss of adherence were studied. Images were acquired on Olympus FLUOVIEW FV1000 confocal laser scanning microscope (excitation: 488 and 543 nm; emission: 505–526 and 612–644 nm, respectively). Quantitative image analysis was performed using FV1000 Viewer-1.7 for fluorescence intensity, and the percent cell death was calculated against total cell population in the respective set.

### MIC determination

The *in vitro* susceptibility of *S. maltophilia* strains to trimethoprim-sulfamethoxazole, minocycline, ciprofloxacin, levofloxacin, ticarcillin-clavulanate, ceftazidime, piperacillin-tazobactam, amikacin, and chloramphenicol was assessed by MIC-Test Strip (Liofilchem; Roseto degli Abruzzi, Italy), according to CLSI guidelines [Clinical Laboratory Standards Institute (CLSI), 2016]. In the case of piperacillin-tazobactam, amikacin, and ciprofloxacin, because no breakpoints are available for *S. maltophilia*, we used those established for *P*. *aeruginosa* [Clinical Laboratory Standards Institute (CLSI), 2016]. *Escherichia coli* ATCC 25922 and *P. aeruginosa* ATCC 27853 were chosen as quality control strains in each batch of tests.

### Statistical analysis

Each test was performed in triplicate and repeated on two different occasions. Statistical analysis was performed using Prism 6 for Windows software (version 6.01; GraphPad Software Inc., La Jolla, USA). Gaussian distribution was evaluated by Kolmogorov–Smirnov-test with Dallal-Wilkinson-Lille for *p*-value. Differences were measured using both parametric (one-way ANOVA-test followed by Tukey's multiple comparison post-test), and non-parametric (Mann–Whitney-test; Kruskal–Wallis ANOVA-test followed by Dunn's multiple comparison post-test) tests. Linear regression analysis was used to assess the significance of a trend. Spearman correlation coefficient was calculated for correlation analysis. MIC-values were considered as discordant for discrepancies ≥ 2 log_2_ concentration steps. Statistical significance was set at 0.05.

## Results

### Bacterial genotyping

**Figure 1:**
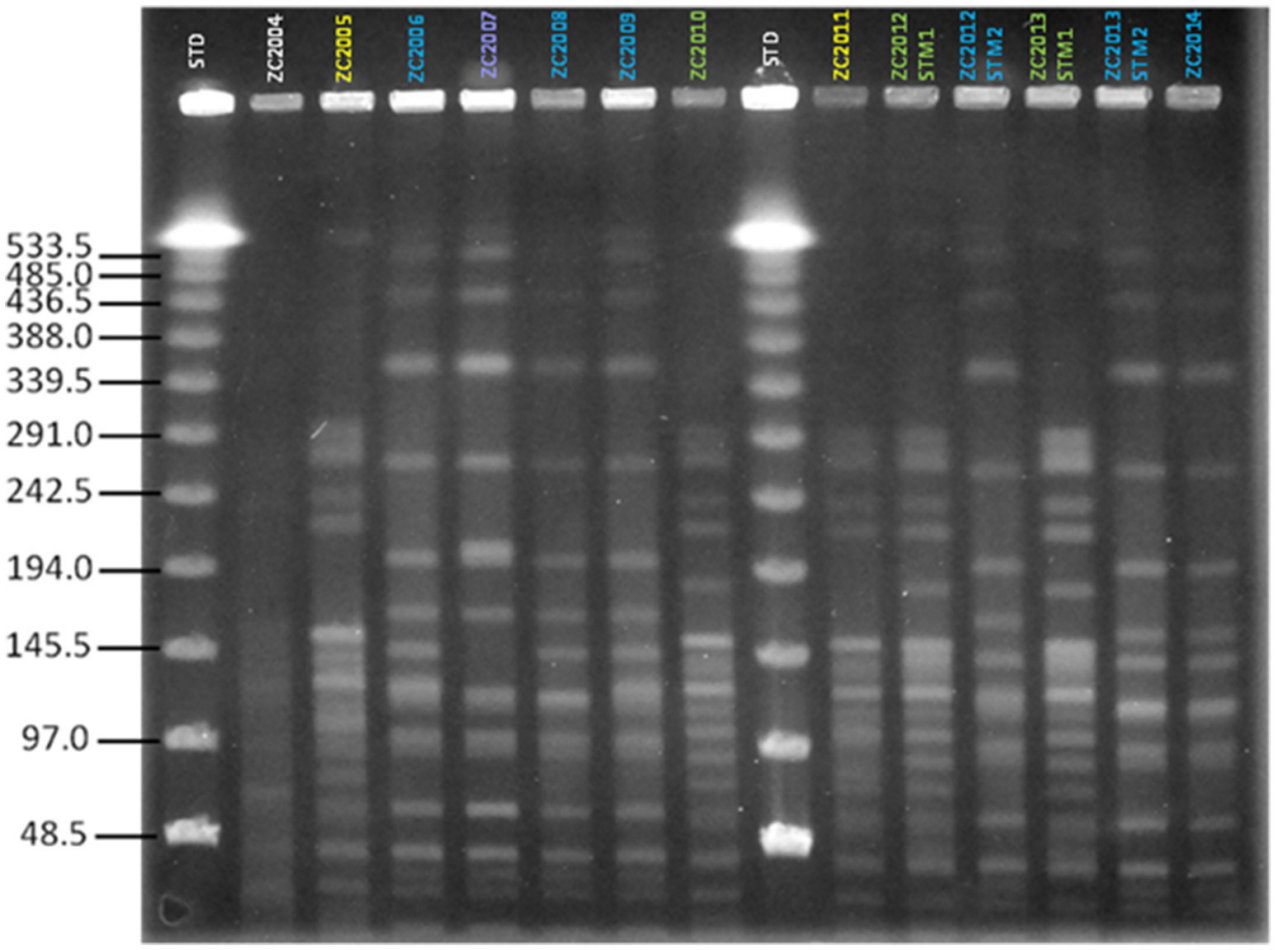
**Clonal relatedness of ***S. maltophilia*** strains, as assessed by PFGE analysis**. The similarity of PFGE profiles was visually assessed, and considered as follows: (i) isolates with identical PFGE patterns were assigned to the same PFGE type and subtype; (ii) isolates differing by one to three bands were assigned to the same PFGE type and were considered genetically related; while (iii) isolates with PFGE patterns differing by more than four bands were considered genetically unrelated and were assigned to different PFGE types. Four pulsotypes were observed: 1.1 (yellow), 1.2 (green), 2.1 (blue), and 2.2 (purple). STD, molecular weight standard. The profile exhibited by ZC2004 strain could not be interpreted because of poor resolution and, consequently, was not assigned to a pulsotype and not enrolled in the study.

We first assessed the clonality of strains isolated from patient ZC at different time points during the course of chronic infection over 11 years. Based on PFGE patterns, two distinct PFGE groups, with four different pulsotypes, were identified among *S. maltophilia* isolates according to the previously described interpretative criteria (Figure 1). PFGE group 1 encompassed two related PFGE subtypes, namely pulsotype 1.1, consisting of two strains (ZC2005 and ZC2011), and pulsotype 1.2, consisting of three strains (ZC2010, ZC2012-STM1, and ZC2013-STM1); PFGE group 2 comprised two related PFGE subtypes, pulsotype 2.1, consisting of six strains (ZC2006, ZC2008, ZC2009, ZC2012-STM2, ZC2013-STM2, and ZC2014); and pulsotype 2.2, consisting of ZC2007 strain only. Strains isolated both in 2012 (ZC2012-STM1 and ZC2012-STM2) and 2013 (ZC2013-STM1 and ZC2013-STM2) belonged to different pulsotypes of unrelated PFGE types. The profile exhibited by ZC2004 strain could not be interpreted because of lack of resolution in the high molecular weight zone of the gel and, therefore, was not further studied.

### Growth rate

**Figure 2:**
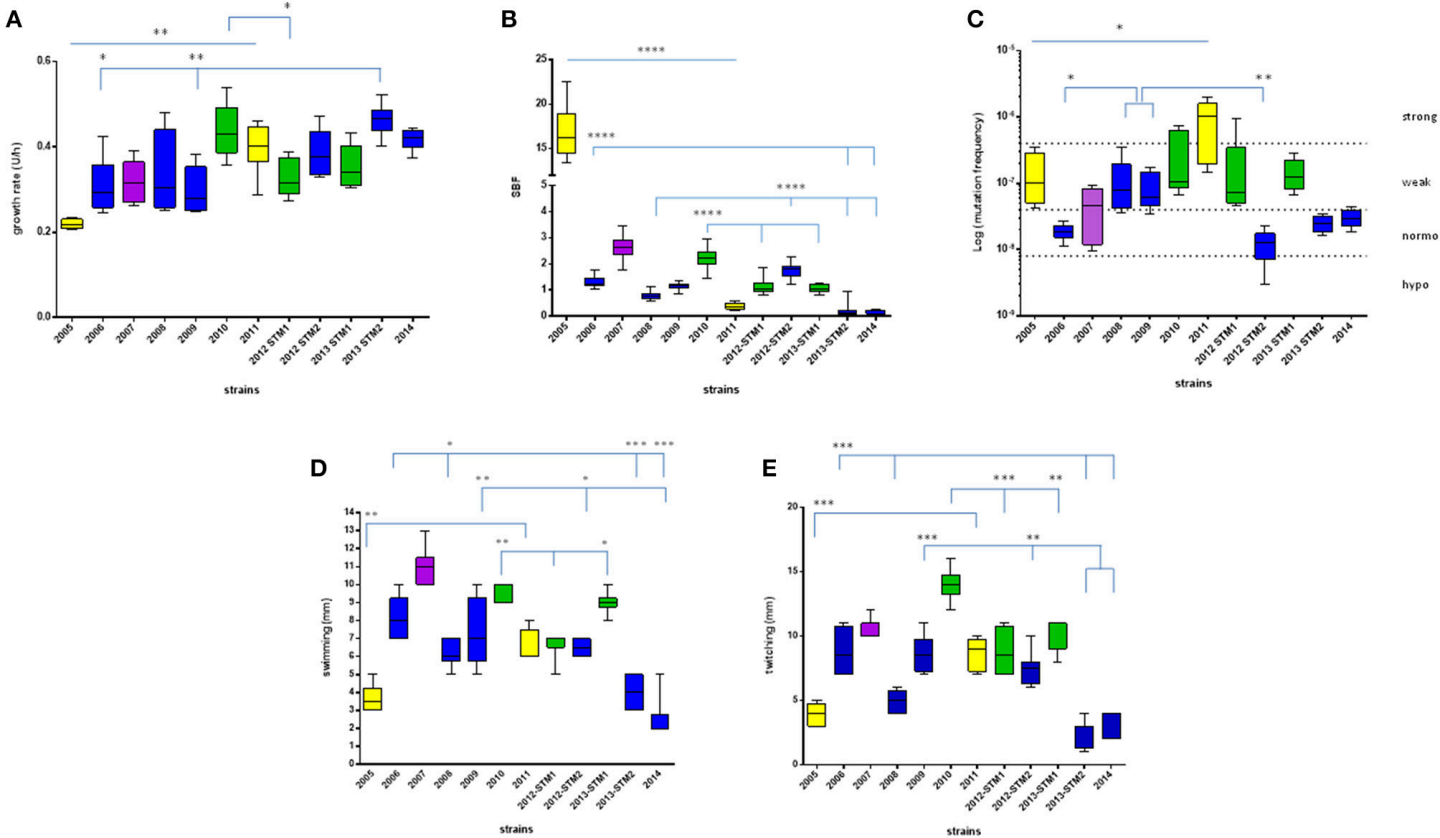
**Phenotypic traits exhibited by ***S. maltophilia*** strains**. Colors indicate the pulsotypes: 1.1 (yellow, *n* = 2 strains), 1.2 (green, *n* = 3 strains), 2.1 (blue, *n* = 6 strains), and 2.2 (purple, *n* = 1 strain). **(A)** Growth rate, expressed as generation time (U/h). **(B)** Biofilm biomass formation, normalized on growth rate and expressed as specific biofilm formation (SBF) index. **(C)** Mutation frequency; strains were classified into four categories based on mutation frequency (*f*): hypo-mutators (*f* ≤ 8 × 10^−9^), normo-mutators (8 × 10^−9^ < *f* < 4 × 10^−8^), weak-mutators (4 × 10^−8^ ≤ *f* < 4 × 10^−7^), and strong-mutators (*f* ≥ 4 × 10^−7^). **(D)** Swimming motility, expressed as diameter of growth zone (mm). **(E)** Twitching motility, expressed as diameter of twitch zone (mm). Results are shown as box and whiskers (*n* = 6, for each strain): the box always extends from the 25th to 75th percentiles, while the line in the middle of the box is plotted at the median. Statistical analysis: ^*^p < 0.05, ^**^p < 0.01, ^***^p < 0.001, ^****^p < 0.0001, Mann–Whitney-test (pulsotype 1.1) or Kruskal–Wallis followed by Dunn's multiple comparison post-test (pulsotypes 1.2 and 2.1).

**Figure 3:**
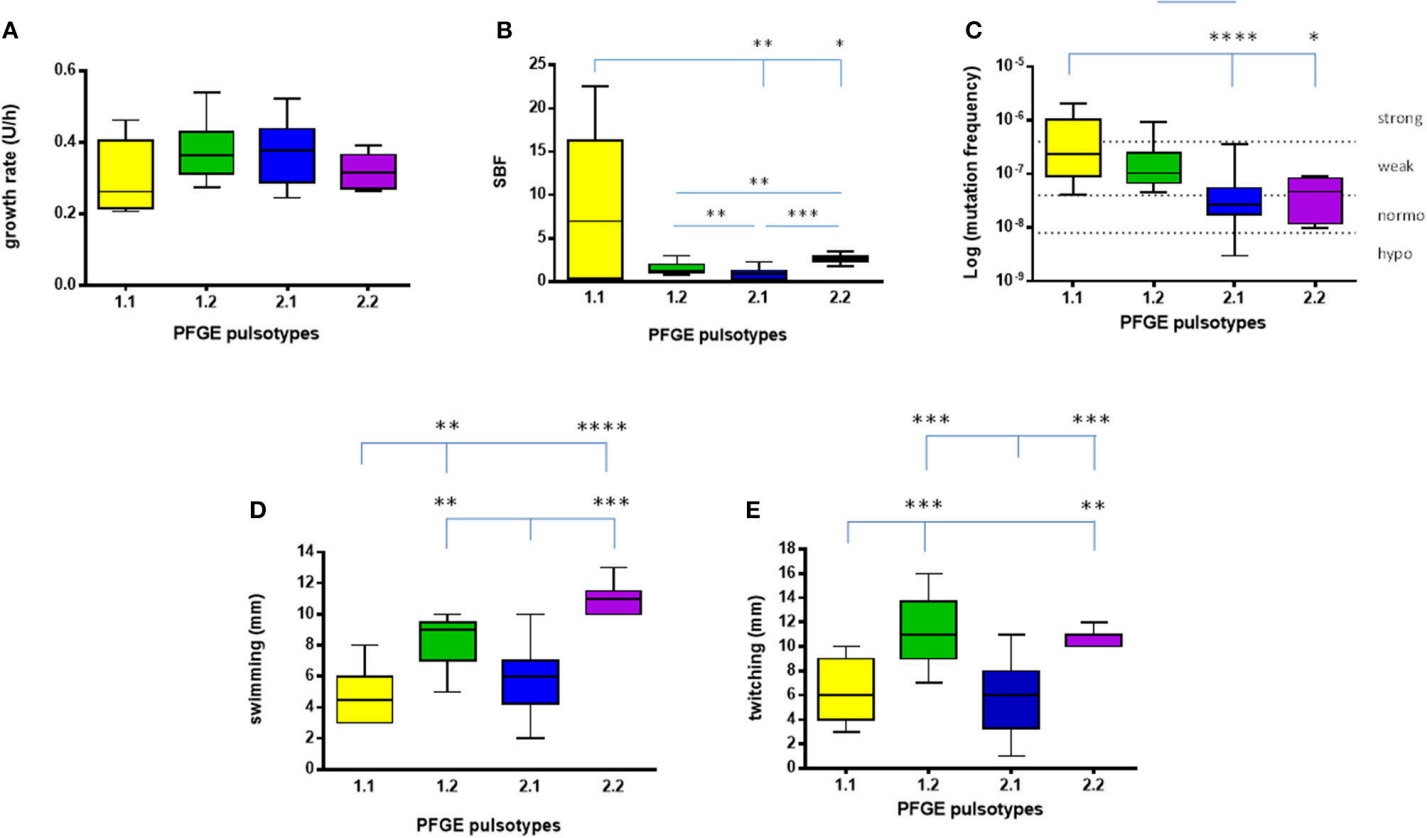
**Phenotypic traits exhibited by ***S. maltophilia*** strains**. Results (*n* = 6, for each strain) are stratified according to the pulsotype: 1.1 (yellow, *n* = 2 strains), 1.2 (green, *n* = 3 strains), 2.1 (blue, *n* = 6 strains), and 2.2 (purple, *n* = 1 strain). **(A)** Growth rate. **(B)** Biofilm biomass formation, normalized on growth rate (SBF; specific biofilm formation). **(C)** Mutation frequency; strains were classified into four categories based on mutation frequency (*f*): hypo-mutators (*f* ≤ 8 × 10^−9^), normo-mutators (8 × 10^−9^ < *f* < 4 × 10^−8^), weak-mutators (4 × 10^−8^ ≤ *f* < 4 × 10^−7^), and strong-mutators (*f* ≥ 4 × 10^−7^). **(D)** Swimming motility. **(E)** Twitching motility. Results are shown as box and whiskers: the box always extends from the 25th to 75th percentiles, while the line in the middle of the box is plotted at the median. Statistical analysis: ^*^p < 0.05, ^**^p < 0.01, ^***^p < 0.001, ^****^p < 0.0001, Kruskal–Wallis followed by Dunn's multiple comparison post-test.

Growth rate values exhibited by each *S. maltophilia* strain were spectrophotometrically assessed, and results are summarized in Figures 2A, 3A.

*S. maltophilia* strains isolated over time significantly differed for growth rate (*p* < 0.0001, Kruskal–Wallis-test), showing a significant upward trend (*p* < 0.01; Table 1).

**Table 1:** *****S. maltophilia*** trends in expression over time across all traits**.

**Pulsotype (*n*)**	**Trait**							
	**Growth rate**	**Biofilm formation**	**Mutation frequency**	**Swimming motility**	**Twitching motility**	***G. mellonella* pathogenicity**	**A549 cells pathogenicity**	**Antibiotic resistance**
1.1 (2)	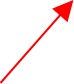	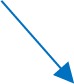	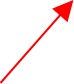	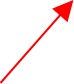	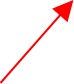	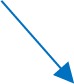	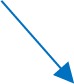	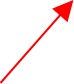
1.2 (3)	____	____	____	____	____	____	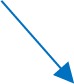	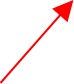
2.1 (6)	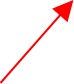	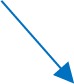	____	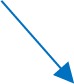	____	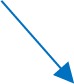	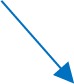	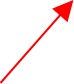
Overall (*n* = 12)	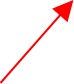	____	____	____	____	____	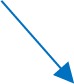	____

The statistical significance of a trend was assessed by Mann–Whitney-test (pulsotype 1.1) or linear regression analysis (pulsotypes 1.2 and 2.1). Pulsotype 2.2 was not considered as composed of one strain only. Arrows indicate significant trends: increased (red) or decreased (blue). Trend in antibiotic resistance was measured by considering mean variation in MIC-values over time, considering only ≥ 4-fold changes.

Significant differences were observed within each pulsotype (Figure 2A). With regard to strains belonging to pulsotype 1.1, ZC2011 showed a growth rate higher compared to ZC2005 (median: 0.402 vs. 0.217, respectively; *p* < 0.01). With regard to pulsotype 1.2, ZC2010 strain showed a median growth rate significantly higher than ZC2012-STM1 (median: 0.429 vs. 0.316, respectively; *p* < 0.05). Among pulsotype 2.1 strains, ZC2013-STM2 grew significantly faster than ZC2006 and ZC2009 strains (median: 0.465 vs. 0.292 and 0.279, respectively; *p* < 0.01).

Considering each pulsotype as a whole, no statistically significant differences were observed (Figure 3A). The kinetics of changes in growth rate showed that both pulsotypes 1.1 and 2.1 significantly increased over the study-period (*p* < 0.0001 and 0.05, respectively), while pulsotype 1.2 remained generally unchanged (Table 1).

### Biofilm formation

The results concerning biofilm biomass formed by each strains tested were normalized on growth rate and expressed as SBF, as shown in Figures 2B, 3B. SBF-values were statistically related to non-normalized biofilm OD_492_-values (data not shown; Spearman *r*: 0.986; *p* < 0.0001).

*S. maltophilia* strains significantly differed for efficacy in forming biofilm (*p* < 0.0001, Kruskal–Wallis + Dunn post-test). Significant differences were found among strains belong to each pulsotype (Figure 2B). With regard to pulsotype 1.1 strains, ZC2005 produced significantly more biofilm compared to ZC2011 (median: 16.19 vs. 0.34, respectively; *p* < 0.0001). Among strains belonging to pulsotype 1.2, ZC2010 formed significantly more biofilm biomass than other strains (median: 2.23 vs. 1.02, and 1.06, respectively, for ZC2010, ZC2012-STM1, and ZC2013-STM1 strains; *p* < 0.0001). With regard to pulsotype 2.1, ZC2012-STM2 produced higher biofilm biomass compared to most of other strains (median: 1.79 vs. 0.76, 1.06, and 0.19; respectively, for ZC2012-STM2, ZC2008, ZC2013-STM2, and ZC2014 strains; *p* < 0.0001).

Pulsotypes significantly differed for biofilm formation (*p* < 0.0001, Kruskal–Wallis-test; Figure 3B). In particular, pulsotype 1.1 produced significantly more biofilm than 2.1 and 2.2 (median: 6.99 vs. 0.93 and 2.63, respectively; *p* < 0.01 and 0.05, respectively), pulsotype 2.2 formed a biofilm biomass significantly higher than 1.2 (median: 1.2; *p* < 0.01) and 2.1 (*p* < 0.001), while pulsotype 1.2 produced higher biofilm amount than 2.1 (*p* < 0.01; Figure 3B).

The kinetics of biofilm biomass formed during the study-period showed a significant downward trend for pulsotypes 1.1 and 2.1 (*p* < 0.0001; Table 1).

### Mutation frequency

Variations in the frequency of mutation exhibited by *S. maltophilia* strains isolated over 10 years are summarized in Figures 2C, 3C.

Mutation frequency significantly differed among *S. maltophilia* strains (*p* < 0.0001, Kruskal–Wallis-test), while no significant trend was observed (Table 1). Significant differences were found among strains belong to each pulsotype (Figure 2C). Among pulsotype 1.1 strains, ZC2005 showed a mutation frequency significantly lower than ZC2011 (median: 9.9 × 10^−8^ vs. 1.0 × 10^−6^, respectively; *p* < 0.05). With regard to pulsotype 2.1, strains ZC2008 and ZC2009 exhibited higher mutation frequency (median: 7.9 × 10^−8^ and 6.2 × 10^−8^, respectively) compared to ZC2006 and ZC2012-STM2 (median: 1.8 × 10^−8^ and 1.2 × 10^−8^, respectively; *p* < 0.05 and 0.01, respectively). Strains belonging to pulsotype 1.2 did not change significantly over the study-period.

According to mutation frequency, most of the strains were weak-mutators (7 out of 12, 58.3%), followed by normo-mutators (4 out of 12, 33.3%), while only one strain resulted to be a strong-mutator (8.4%). No hypo-mutators were found (Figure 2C).

Considering the pulsotypes as a whole, pulsotype 1.1 showed higher frequency compared to pulsotype 2.1 and 2.2 (median: 2.3 × 10^−7^ vs. 2.7 × 10^−8^, and 4.6 × 10^−8^, respectively; *p* < 0.0001 and 0.05, respectively; Figure 3C). The only hyper-mutator strain belonged to pulsotype 1.1, while pulsotype 1.2 consisted of weak-mutators only. The strains belonging to pulsotype 2.1 were mainly normo-mutators (66.6%), while the remaining ones were weak-mutators. No significant changes were observed among strains belonging to each pulsotype.

The kinetics of the median mutation frequency showed that in pulsotype 1.1 only strains increased (*p* < 0.05) their mutation frequency over the study-period, shifting from weak (ZC2005)- to strong (ZC2011)-mutator phenotype. No particular trend was observed for other pulsotypes (Table 1).

### Motility

Swimming and twitching motility levels exhibited by *S. maltophilia* strains are summarized in Figures 2D,E, 3D,E, respectively. None of strains showed swarming motility.

Significant differences were found among strains both for swimming and twitching (*p* < 0.0001). Particularly, the motility exhibited by ZC2011 strain was significantly higher compared to that of ZC2005 (swimming, median: 6.0 vs. 3.5 mm, respectively; *p* < 0.01; twitching, median: 9 vs. 4 mm, respectively; *p* < 0.001). With regard to pulsotype 2.1, the motility observed for ZC2006 strain was significantly higher than ZC2008, ZC2013-STM2, and ZC2014 strains (swimming: 8 vs. 6, 4, and 2 mm, respectively; *p* < 0.05; twitching: 8.5 vs. 5, 3, and 2 mm, respectively; *p* < 0.001).

Among strains belonging to pulsotype 1.2, ZC2012-STM1 strain showed a swimming motility significantly lower than strains ZC2010 and ZC2013-STM1 (7 vs. 10 and 9 mm, respectively; *p* < 0.05; Figure 2D). Contrarily, twitching motility was significantly higher in ZC2010 strain, compared to ZC2012-STM1 and ZC2013-STM1 strains (14.0 vs. 8.5, and 9 mm, respectively; *p* < 0.001; Figure 2E).

With regard to each pulsotype, a similar trend was found for both swimming and twitching motilities (Figures 3D,E). In particular, a comparable trend for both swimming and twitching motilities was observed for the strains belonging to pulsotypes 1.1 and 2.1.

Pulsotype 1.1 showed significantly lower motility, compared to pulsotypes 1.2 and 2.2 (swimming: 4.5 vs. 9, and 11, respectively; *p* < 0.01; twitching: 6 vs. 11 and 11 mm, respectively; *p* < 0.01), whereas motility exhibited by pulsotype 2.1 (6 mm, for both swimming and twitching) was significantly lower compared to pulsotypes 1.2 and 2.2 (*p* < 0.01).

The kinetics of changes in swimming and twitching motilities over the study-period showed a significant upward trend in pulsotype 1.1 (*p* < 0.01), whereas a trend toward decreased swimming motility was found in pulsotype 2.1 strains (*p* < 0.05; Table 1).

### Virulence assays

**Figure 4:**
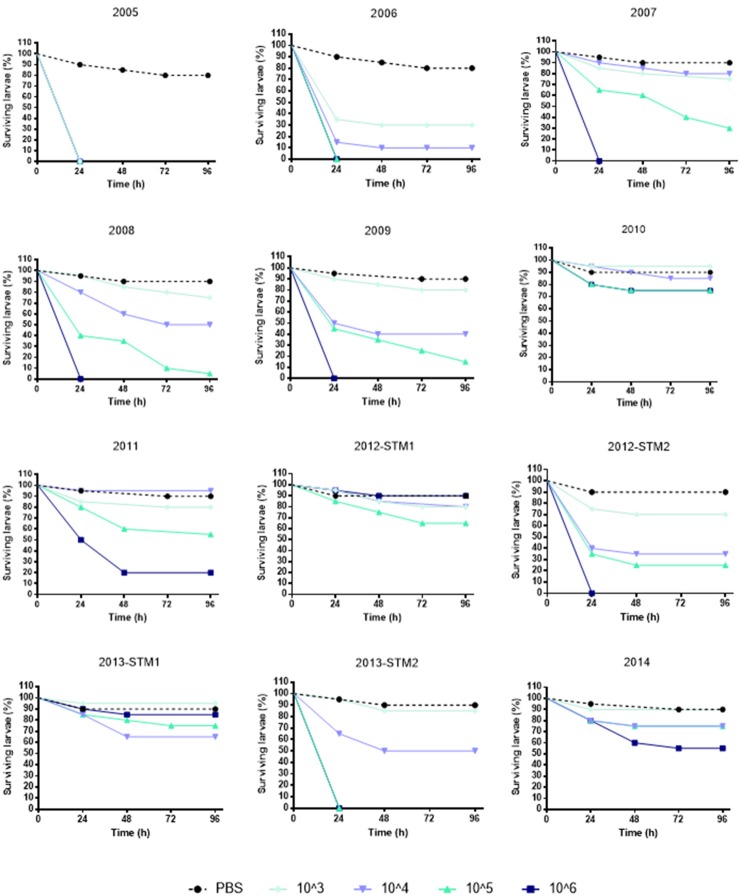
**Survival of ***Galleria mellonella*** over 96 h following infection**. Each strain was used at the following infective doses prepared in PBS: 10^6^, 10^5^, 10^4^, and 10^3^ CFU/larva. Uninfected control larvae were exposed to PBS only (black dotted line). Larvae were incubated at 37°C for 96 h and checked daily for survival, considering dead those not reactive to touch. Results are shown as mean + *SD*.

(i) *G. mellonella* infection assay. The kinetics of *G. mellonella* survival monitored over 96 h following infection with *S. maltophilia* showed that the killing activity was generally dose-dependent, regardless of strain or pulsotype considered (Figure 4).

To comparatively evaluate the pathogenicity of tested strains, a “pathogenicity score” was assigned to each strain (Table 2). ZC2005 was the most virulent strain (score: 8), causing the killing of all infected larvae already at 24 h and at the lowest dose used (10^3^ CFU/larva). Other strains showed striking differences in virulence, except for ZC2010, ZC2012-STM1, ZC2013-STM1, and ZC2014 strains that resulted to be not virulent (score: 1), not being able to kill at least 50% of infected larvae following 96 h-exposure to the highest dose (10^6^ CFU).

**Table 2:** **Pathogenicity of 12 ***S. maltophilia*** strains, isolated from the same CF patient over 10 year-period, as assessed in ***G. mellonella*****.

**Strains**	**Time (h) required to obtain LD_50_ at the following infective doses (CFU/larva)**				**Pathogenicity score**
	**10^3^**	**10^4^**	**10^5^**	**10^6^**	
ZC2005	< 24	< 24	< 24	< 24	8
ZC2006	24	24	< 24	< 24	7
ZC2007			72	< 24	3
ZC2008		72	24	< 24	4
ZC2009		24	24	< 24	6
ZC2010					1
ZC2011				24	2
ZC2012-STM1					1
ZC2012-STM2		24	24	< 24	6
ZC2013-STM1					1
ZC2013-STM2		48	< 24	< 24	5
ZC2014					1

Each strain was inoculated, at different doses (10^3^, 10^4^, 10^5^, and 10^6^ CFU/larva), in 20 larvae. Following infection, the larvae mortality was assessed at different times (24, 48, 72, and 96 h), and LD_50_ (the dose required to kill 50% of the infected larvae) was recorded. For each strain, the “pathogenicity score” was calculated assigning decreasing values from the highest (score: 8) to the lowest (score: 1) virulence, as assessed considering both dose and time necessary to obtain LD_50_. In some cases, 24 h-exposure to S. maltophilia caused the killing of all larvae (LD_50_ < 24 h). Pulsotypes are indicated by colors: 1.1 (yellow), 1.2 (green), 2.1 (blue), and 2.2 (purple).

Pulsotypes 1.1 and 2.1 showed comparable virulence (mean score: 5, and median score: 5.5, respectively), significantly higher than pulsotype 1.2 (median score: 1).

**Figure 5:**
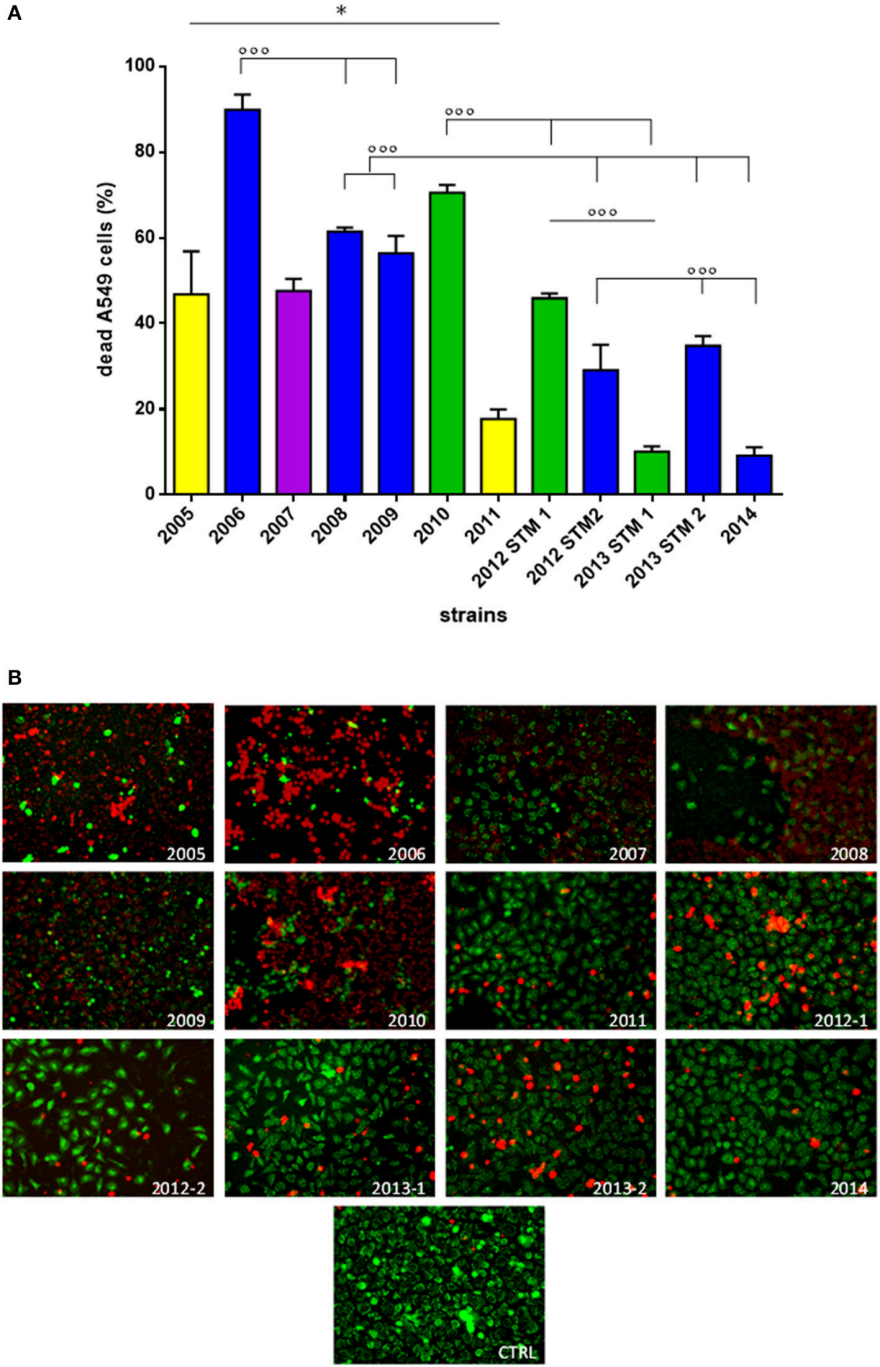
**Effect of ***S. maltophilia*** exposure on human A549 alveolar cells**. Cell monolayers were exposed for 24 h to each *S. maltophilia* strain at MOI 500. The co-culture sets were washed, and then stained with Live/Dead^TM^ assay. Images were acquired by confocal laser scanning microscope, and quantitative image analysis was performed for fluorescence intensity. **(A)** The percent cell death was calculated compared to uninfected control samples (CTRL; cell death, 0%). Bar colors indicate the pulsotypes: 1.1 (yellow, *n* = 2 strains), 1.2 (green, *n* = 3 strains), 2.1 (blue, *n* = 6 strains), and 2.2 (purple, *n* = 1 strain). Results are expressed as mean + *SD* (*n* = 6). ^*^p < 0.05, unpaired *t*-test; ^◦◦◦^*p* < 0.001, ANOVA followed by Tukey's multiple comparison post-test. **(B)** CLSM micrographs of infected A549 cell monolayers, stained with Syto-9 (green fluorescence, indicating live cells), and propidium iodide (red fluorescence, indicating dead cells). Representative microscopic fields are shown. Magnification, 100x.

The same trend in virulence was observed, over time, in strains belonging to pulsotypes 1.1 and 2.1: pathogenicity score in fact significantly decreased from 8 (ZC2005) to 2 (ZC2011), and from 7 (ZC2006) to 1 (ZC2014), respectively. No change was observed in pulsotype 1.2 strains (Table 1).

(ii) A549 cells co-culture assay. *S. maltophilia* pathogenicity was also assessed on human A549 alveolar cells using Live/Dead™ cell viability staining (ThermoFisher Scientific; Figure 5).

Generally, strains significantly differed for pathogenicity level (*p* < 0.0001), although a downward trend was observed over the time (Figure 5A; Table 1). With regard to pulsotype 1.1, the damage caused by ZC2005 strain was significantly higher compared to that of ZC2011 (mean percentage ± *SD*: 46.7 ± 10.0 vs. 17.7 ± 2.1, respectively; *p* < 0.05). Among pulsotype 1.2 strains, damage significantly decreased from ZC2010 toward 2012-STM1 and ZC2013-STM1 (mean ± *SD*: 70.4 ± 1.8, 45.8 ± 1.1, and 10.0 ± 1.2, respectively; *p* < 0.001). Striking significant differences were also found among strains belonging to pulsotype 2.1, where pathology rate significantly decreased over time from 89.8 ± 3.5 (ZC2006) to 9.1 ± 1.9 (ZC2014) (*p* < 0.001). Linear regression analysis confirmed the existence of a negative trend in each pulsotype (Table 1).

No significant differences were found in cellular damage among pulsotypes.

However, subtler differences between the temporal profiles within a pulsotype were observed with regard to non-lethal effects, including cell rounding and detachment (Figure 5B). Cells exposed to ZC2010 exhibited unusually high rounding comparable to ZC2006, whereas ZC2008 consistently showed high detachment of the epithelial monolayer. The early colonizers like ZC2005 provoked all three effects, whereas ZC20014 strain caused minimal damage.

A positive, although statistically not significant, trend was observed between *G. mellonella* and A549 assays, considering results either as a whole (Spearman *r*: 0.395) or stratified to pulsotypes 1.1 and 2.1 (Spearman *r*: 0.550).

### Antibiotic susceptibility

**Figure 6:**
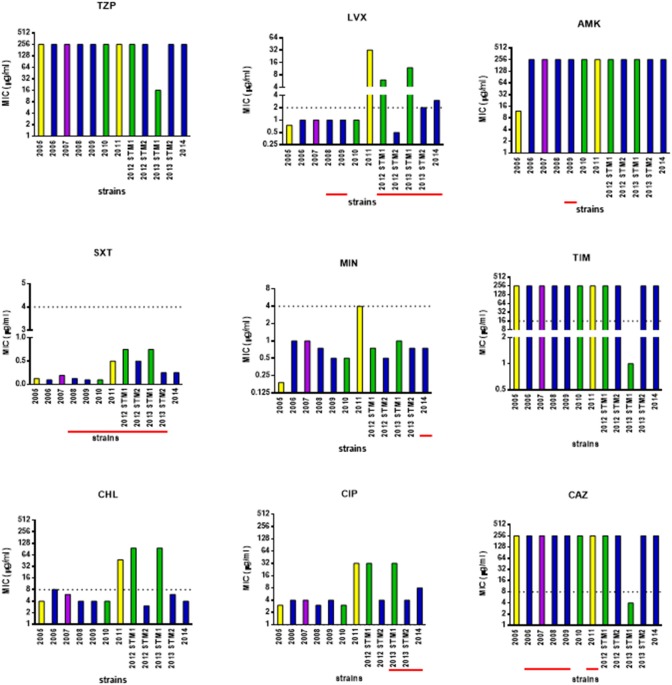
**Antimicrobial susceptibility of ***S. maltophilia*** strains**. Bar colors indicate the pulsotypes: 1.1 (yellow, *n* = 2 strains), 1.2 (green, *n* = 3 strains), 2.1 (blue, *n* = 6 strains), and 2.2 (purple, *n* = 1 strain). MIC-values were determined, using the MIC-test strip method, for the following antibiotics: piperacillin/tazobactam (TZP), levofloxacin (LVX), amikacin (AMK), cotrimoxazole (SXT), minocycline (MIN), ticarcillin/clavulanate (TIM), chloramphenicol (CHL), ciprofloxacin (CIP), and ceftazidime (CAZ). The dotted line indicates the breakpoint MIC for susceptibility [Clinical Laboratory Standards Institute (CLSI), 2016], when available. Underlined in red, the years when the antibiotic was therapeutically administered to patient. Information on antibiotic therapy was available for all years but 2005.

The susceptibility patterns of the sequential *S. maltophilia* strains under study were determined by the MIC-test strip method, and results are shown in Figure 6.

Considering the strains as a whole, MIC of each antibiotic significantly varied over the study-period: 16 to ≥256 μg/ml (piperacillin-tazobactam), 0.5 to ≥32 μg/ml (levofloxacin), 12 to ≥256 μg/ml (amikacin), 0.094–0.75 (trimethoprim-sulfamethoxazole), 0.19–4 μg/ml (minocycline), 1 to ≥256 μg/ml (ticarcillin-clavulanate), 3–96 μg/ml (chloramphenicol), 3 to ≥32 μg/ml (ciprofloxacin), and 4 to ≥256 μg/ml (ceftazidime).

Pulsotype 1.1 strains significantly increased their MIC for levofloxacin (from 0.75 to 32 μg/ml), amikacin (from 4 to 32 μg/ml), cotrimoxazole (from 0.94 to 0.5 μg/ml), minocycline (from 0.19 to 4 μg/ml), chloramphenicol (from 4 to 48 μg/ml), and ciprofloxacin (from 3 to 32 μg/ml), shifting toward resistant class in the case of chloramphenicol. The mean increase in MIC-values over time was 18.1-fold.

MIC-values exhibited by the strains belonging to pulsotype 1.2 significantly increased over the study-period, in the case of levofloxacin (from 1 to 12 μg/ml), chloramphenicol (from 4 to 96 μg/ml), and ciprofloxacin (from 3 to 32 μg/ml). This resulted in susceptible-to-resistant transition in the case of levofloxacin and chloramphenicol, but not for ciprofloxacin whose MICs always indicated resistance. In contrast, MICs significantly decreased of at least 80-fold for ceftazidime and ticarcillin/clavulanate (from 250 to 3 μg/ml, and from 256 to 1 μg/ml, respectively), and of 16-fold for piperacillin-tazobactam (from 256 to 16 μg/ml), switching in all cases from resistant to susceptible class. Trimethoprim-sulfamethoxazole MIC increased as well, although the range was within susceptibility breakpoint (from 0.094 to 0.75 μg/ml). The mean increase in MIC-values over time was 13.6-fold.

Strains belonging to pulsotype 2.1 exhibited increased MICs for levofloxacin only, passing from susceptible to resistant class (from 1 to 3 μg/ml). The mean increase in MIC-values over time was 2.8-fold.

The only hyper-mutator strain ZC2011 showed the highest number of antibiotic resistances. Interestingly, a trend toward multiple antibiotic resistance among hyper- (4), weak- (2.6 ± 0.97), and normo-mutator (2.3 ± 0.5) strains was noted, although this was not statistically significant.

Mutator phenotypes showed higher mean MIC-values compared to non-mutator phenotype in the case of levofloxacin (mean ± *SD*: 1.6 ± 1.1 vs. 6.8 ± 10.9, respectively), chloramphenicol (mean ± *SD*: 5.3 ± 2.2 vs. 32.8 ± 41.8, respectively), and ciprofloxacin (mean ± *SD*: 5.0 ± 2.0 vs. 14.1 ± 14.8, respectively). However, these differences were not statistically significant probably due to high *SD*-values.

In pulsotype 2.1 strains, increased antibiotic resistance was observed against fluoroquinolones following antibiotic treatment cycles with levofloxacin or ciprofloxacin.

### Trends in *S. maltophilia* adaptive phenotypes

The phenotypic traits significantly changed in each *S. maltophilia* pulsotype over the study-period are summarized in Table 1.

The pattern of evolution followed by *S. maltophilia* was dependent on pulsotype considered. Following long-lasting *S. maltophilia* infection in CF lung, strains belonging to pulsotype 1.1 resulted to be the most adapted, being significantly changed in all traits considered. Pulsotype 2.1 strains showed variations in all traits but mutation frequency and twitching motility, sharing with pulsotype 1.1 the same trend for growth rate, biofilm formation, pathogenicity, and antibiotic resistance, while an opposite one was observed for swimming motility. All pulsotypes were affected in A549 pathogenicity and antibiotic susceptibility. The same temporal trends were confirmed using non- normalized biofilm OD_492_-values (data not shown).

**Figure 7:**
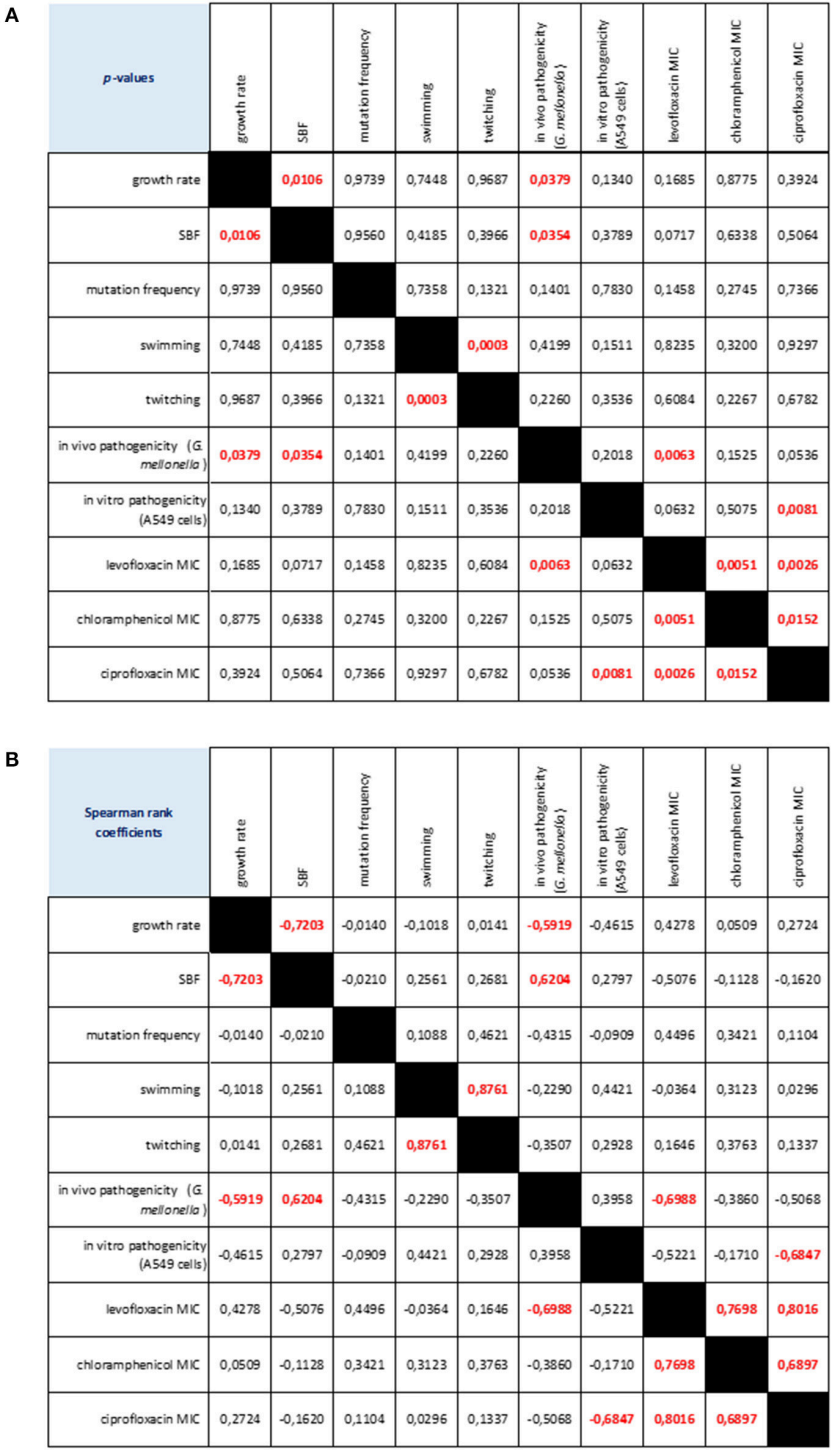
**Correlation matrix of phenotype-phenotype associations, as determined by Spearman rank correlation coefficient**. **(A)** *p*-values: red values in bold indicate a significant (*p* < 0.05) correlation between any given phenotype pair. **(B)** Spearman rank coefficients. Red values in bold indicate a significant (*p* < 0.05) correlation between any given phenotype pair: positive and negative values indicate direct and inverse correlations, respectively.

Considering the strains as a whole, we found several relationships among the phenotypic traits considered (Figure 7). Swimming and twitching motilities were positively correlated (Spearman *r* = 0.876; *p* < 0.001), whereas a negative correlation was observed between growth rate and biofilm formation (Spearman *r* = −0.720; *p* < 0.05). Pathogenicity, as assessed in *G. mellonella* model, was negatively correlated both with growth rate (Spearman *r* = −0.591; *p* < 0.05) and levofloxacin MIC (Spearman *r* = −0.698; *p* < 0.01), whereas a positive association was found with biofilm formation (Spearman *r* = 0.624; *p* < 0.05). The mortality observed in A549 cells was negatively associated with ciprofloxacin MIC (Spearman *r* = −0.684; *p* < 0.01). Susceptibility to levofloxacin, ciprofloxacin and chloramphenicol were positively correlated each other.

## Discussion

In contrast to *P. aeruginosa* CF lung colonization, where genetic adaptations leading to phenotypic variation are well known (Clark et al., 2015), the adaptive characters in *S. maltophilia* that drive its persistence in CF lungs is largely unknown. It is, therefore, interesting to understand trait changes during *S. maltophilia* persistence in CF lungs and to evaluate whether there is a typical phenotypic profile related to chronic infection. With that aim, the approach followed in this study was to combine genotypic profiling with phenotypic characterization to compare sequential *S. maltophilia* isolates recovered from a chronically infected CF patient.

The main conclusion arising from our results is the evidence that during the course of chronic lung colonization *S. maltophilia* develops new phenotypes and modulates its pathogenicity, probably reflecting genetic or epigenetic changes.

*S. maltophilia* population structure within CF patient airway is highly dynamic during long-term chronic infection. Our findings in fact showed that 2 distinct PFGE genotypes, with 4 different pulsotypes, colonize CF lung over time, and multiple lineages are present at the same time, as observed during years 2012 and 2013. Further, we observed replacements of infecting genotypes that, from an ecological perspective, could suggest the existence of a competition among different genotypes for the same specific niches of the CF patient lung. The patient harbored pulsotypes significantly different for isolation frequency over time. A similar high degree of strain diversity was found by Vidigal et al. (2014) in *S. maltophilia* consecutively isolated from chronically colonized CF patients. A more diverse community reflects niche separation within the host making bacterial subpopulations better able to resist an external stress such as exposure to antibiotics or the human defense system. Our results cannot provide information if strains come from an environmental source rather than acquired from another patient, although we recently found that both scenarios are plausible (Pompilio et al., 2011).

Growth rate is an accepted measure of adaptation and has been previously used to evaluate fitness deficits associated with antibiotic resistance (Pope et al., 2008). It has been suggested that diminished growth rate of *P. aeruginosa* in the sputum of chronically infected CF patients is due to the low PMN-related availability of O_2_ within the mucus (Kragh et al., 2014) or, alternatively, to genetic adaptations (Rau et al., 2010). In contrast, we observed that *S. maltophilia* growth rate, as calculated under our experimental setting (TSB medium, 37°C, static conditions), increased over time, considering strains both as a whole and stratified on pulsotype. Our findings suggest that *S. maltophilia*, contrarily to *P. aeruginosa*, might predominantly colonize respiratory zone (oxygenated due to continuous O_2_ supply from the venous blood passing alveoli), rather than the anoxic infectious mucus in the bronchi of the conducting zone. We also found that growth rate negatively correlated with biofilm formation. This is consistent with the finding that slow bacterial growth enhances extracellular polymeric substance matrix production, therefore allowing stratification of the bacterial community to form biofilms (Sutherland, 2001). However, future studies are needed to evaluate if *S. maltophilia* fitness could be dependent on medium and conditions used.

The ability of *S. maltophilia* cells to form biofilm was reduced during the late stages of a chronic infection. We recently observed a reduced efficiency in forming biofilm by *S. maltophilia* CF isolates, compared to non-CF ones, probably secondary to the bacterial adaptation to a stressed environment such as CF lung (Pompilio et al., 2011). Similarly, *P. aeruginosa* isolates from chronically infected patients are often impaired in forming biofilms (Head and Yu, 2004).

Conditions in CF airways consistently select against bacterial functions deemed essential for biofilm formation during *in vivo* bacterial evolution (Wilder et al., 2009). At early stage of disease, it is more suitable to increase biofilm formation to gain benefits including superior access to nutrients and resistance to environmental insults, such as phagocytosis and antibiotic treatment. During long-term persistence in the airways of CF patients, the increased lung damage, a higher prevalence of co-colonizing pathogens, and increase of neutrophils make necessary to impair biofilm formation to disseminate in new, ecologically more favorable, airways locations (Nadell and Bassler, 2011; Steenackers et al., 2016).

The adaptation of bacterial population to new or challenging environments normally results in spontaneous generation of hypermutable strains which display higher mutation frequencies than their normal counterparts, as a result of defects in the DNA repair system or proof reading systems. Antibiotics—as well as host environment—select for these variants, as these undergo more genetic mutations and are better able to adapt and survive under the antimicrobial pressures *in vivo* (Rodriguez-Rojas et al., 2013).

In CF lung the selection for hypermutable *P. aeruginosa* strains becomes more frequent in later stages or chronic infection in CF patients, suggesting that genetic and phenotypic diversification plays an essential role in the adaptation of *P. aeruginosa* to the hostile and diverse CF lung environment, probably by selecting for less virulent phenotypes (Hogardt et al., 2007; Oliver, 2010).

Interestingly we did not observe hypermutability in our panel of *S. maltophilia* strains. Half of *S. maltophilia* strains we tested were in fact weak mutators, while only one strain (8.4%) was hypermutator. These observations are similar to that reported by O'Neill and Chopra (2002) in *S. aureus* clinical isolates, but contrary to findings by Vidigal et al. (2014) who observed comparable frequency of strong- (31.2%) and weak-mutators (27.7%), and a lower frequency of hypomutators (17.7%), in 90 *S. maltophilia* isolates collected from the sputum of 19 CF patients considered chronically colonized. However, in agreement with this study, we found that mutation rates of the most clonally related genotypes varied over time with the tendency to become less mutable, except for pulsotype 1.1 that significantly increased mutation frequency over time. We are tempted to hypothesize that mutation frequency does not contribute significantly to the adaptation of *S. maltophilia* population to CF lung. However, this discrepancy could be also due to the small number of strains we tested, therefore warranting further studies on larger populations.

The bacterial colonization of CF airways is mediated by the adhesion of cell appendages such as flagellum and type IV pili to host epithelial cell surface. Our findings revealed that in *S. maltophilia* motility changes during long-term colonization depend on the pulsotype considered. Swimming motility is well described as an adaptive trait in *P. aeruginosa* infections in CF whereby in contrast to initially infecting motile strains, chronic ones are characterized by the lack of swimming motility due to the loss of the flagellum (Huse et al., 2013). We observed a similar trend for pulsotype 2.1 strains whose swimming motility significantly decreased over study-period. The potential reason for this phenotypic selection *in vivo* is that the decreased flagellar motility may enable *S. maltophilia* to better evade immune recognition and airway clearance by phagocytosis. Several studies have in fact shown the inability of macrophages to phagocytose non-flagellated *P. aeruginosa* isolates (Mahenthiralingam et al., 1994), and the reduced inflammasome activation and antibacterial IL-1β host response following the loss in motility (Patankar et al., 2013).

Inversely, strains belonging to pulsotype 1.1 significantly increased swimming and twitching motilities during chronicization. In agreement with our findings, *Burkholderia cenocepacia* complex isolates from chronic infections were found not to lose swimming motility (Zlosnik et al., 2014), even showing increased expression in genes associated with flagella assembly and adhesion during the late stage of infection (Mira et al., 2011). We do not know the significance of this observation, although this trend could be due to the small number of strains (*n* = 2) belonging to pulsotype 1.1.

In partial agreement with our previous findings (Pompilio et al., 2011), swimming and twitching motility were positively correlated, but neither associated with biofilm formation. Although it is generally agreed that motility and biofilm development are mutually exclusive events (Belas, 2013), flagella are not only required as a mechanical device for propulsion, but also play a critical role in the initial stages of surface adhesion that leads to the formation of a biofilm, therefore representing attractive therapeutic targets (Erhardt, 2016).

Accumulating evidences support that *S. maltophilia* exhibits plethora of pathogenic determinants to exert its association with human respiratory epithelium. These determinants are unique in context of this pathogen's limited virulence that limits its invasive potential in comparison to other evolved pathogens like *P. aeruginosa*. However, *S. maltophilia* is increasingly known to employ multifactorial determinants like extracellular proteases (Karaba et al., 2013), host cell actin modifiers (MacDonald et al., 2016), quorum signaling molecules (Huedo et al., 2015), and highly evolved efflux-pumps (Chang et al., 2015), which independently or together offer formidable recalcitrance and pathogenic fitness.

*P. aeruginosa* adaptation in CF airways selects patho-adaptive variants with a strongly reduced ability to cause acute infection processes in a host-independent way (Hoboth et al., 2009; Folkesson et al., 2012; Lorè et al., 2012). Consistent with these findings, our data clearly showed that the virulence potential of *S. maltophilia* plays little if any role in its ability to persist in CF airways. Pathogenicity, as measured with similar trends both in *G. mellonella* and human lung epithelial cells, was in fact severely reduced over time. This result indicates a host-pathogen relationship that results in attenuated virulence and pathogenicity during the establishment of chronic infection. Virulence factors or determinants are in fact often non-essential to the pathogen and, consequently, are lost (Brown et al., 2012). Further studies are needed to evaluate whether reduced virulence in *S. maltophilia* is itself adaptive in terms of helping bacterial cells to go unnoticed by host immune system (Gama et al., 2012) or resist antibiotic therapy (Malone et al., 2010), or if it is a pleiotropic cost associated with other within-host adaptation.

However, despite decreased virulence *S. maltophilia* might retain the ability to contribute to disease pathogenesis in CF lung by inducing high proinflammatory cytokine and adhesion molecule expression, as described in *P. aeruginosa* (Hawdon et al., 2010). In this regard, we observed that *S. maltophilia* biofilm formation efficiency, although decreased over time, is directly associated with mortality rate in *G. mellonella*, a finding supported elsewhere for *Candida albicans* and *Cryptococcus neoformans* (Cirasola et al., 2013; Benaducci et al., 2016).

Chronic respiratory infections by *S. maltophilia* are very difficult to treat due to bacterial intrinsic resistance to a wide number of antibiotics, and ability to develop high-level resistance during antibiotic treatment and to adapt to and resist other adverse environmental conditions (Ryan et al., 2009; Chang et al., 2015). Although we observed variable resistance profiles along the study period, as a general trend evolution toward lower levels of susceptibility to antibiotics was observed over time, in terms of mean increase in MIC-values and accumulation of resistances. Interestingly, in the case of levofloxacin and chloramphenicol, MIC changes even resulted in susceptible-to-resistant category transition. All strains were susceptible to cotrimoxazole and mynocycline, although a genotype-dependent trend toward higher MIC-values was observed over time for both antibiotics. Our findings could have significant implications in the management of CF patients since these drugs are considered first-line therapeutic choices for *S. maltophilia* infections (Wei et al., 2016).

Fluoroquinolones are commonly used to treat infections due to *S. maltophilia*. However, their overuse worldwide has resulted in higher resistance rates in many kinds of pathogenic bacteria, including *S. maltophilia* (Pien et al., 2015). In this respect, following administration of parental or inhaled fluoroquinolones therapy reduced susceptibility to both ciprofloxacin and levofloxacin was observed in pulsotype 2.1 strains. The ability of *S. maltophilia* to develop resistance during antibiotic treatment is in agreement with the generalized idea that this adaptive mechanism is among the important features contributing to persistent infection.

No correlation could be established between antibiotic resistance and the amount of the biofilm formed, indicating that other relevant mechanisms might also contribute to the increased resistance registered toward several antimicrobials of different classes. In this regard, contrarily to Vidigal et al. (2014), we found higher MIC-values in mutator strains compared to non-mutator ones. A correlation between *S. maltophilia* mutators and increasing antibiotic resistance was found namely for ciprofloxacin, levofloxacin, and chloramphenicol. It is thought that the genetic and phenotypic changes that confer resistance also result in concomitant reductions in *in vivo* virulence (Cameron et al., 2015). For the first time, the present work described in *S. maltophilia* a direct relationship between the development of resistance to fluoroquinolones and reduced pathogenicity.

Taken together, our results show that *S. maltophilia* is a versatile pathogen which can adapt successfully to a highly stressful environment such as CF lung. To this, *S. maltophilia* pays a “biological cost,” as suggested by the presence of relevant genotypic and phenotypic heterogeneity within a bacterial population chronically infecting the CF lung. A number of social traits are in fact changed over time, probably as a result of evolution within a lineage, or by displacement of one by another lineage. Although adaptation occurred with selection of substantially different *S. maltophilia* phenotypes, depending on genotype considered, it was possible to detect a general trend of adaptation toward less virulence and increased antibiotic resistance in our investigated isolates.

*S. maltophilia* adaptation, measured as number of changed traits, is associated with length of persistence. In addition, the establishment of a highly heterogeneous bacterial population, suggestive for niche separation in the host by different strains, indicates that populations are significantly more complex and dynamic than can be described by the analysis of any single isolate and can fluctuate rapidly to changing selective pressures.

Although the differences at the genetic or epigenetic level giving rise to phenotypic variability in CF isolates are not yet known, our results gained new insights into the behavior of *S. maltophilia* during persistence in CF lung that will hopefully help to identify vulnerabilities and potential targets for the development of treatment strategies directed at chronic infection.

The main limitation of the present study is that having considered only one chronically infected patient does not allow us to evaluate if the adaptation process may relate to the complexity of the individual host niche. In future investigations, we plan to expand the number of patients in order to: (i) study the precise microenvironmental pressures driving diversification we observed among phenotypic traits within *S. maltophilia* populations in the CF lung; (ii) identify specific genetic determinants contributing to such diversity, by using of whole genome sequencing of large numbers of isolates coupled with phenotypic characterization and genome-wide association analyses; and (iii) evaluate other phenotypic adaptations classically involved during progression from acute to chronic infection (i.e., exopolysaccharide production, quorum sensing, expression of virulence factors associated with chronic infection).

## Author contributions

AP, VC, DG, MC, GG, and LV performed analyses. EF collected and processed clinical specimens, and provided clinical expertise for discussion of results. AP, DG, and GD statistically evaluated results, drafted the manuscript and defined the study design. All authors read, reviewed, and approved the final manuscript.

### Conflict of interest statement

The authors declare that the research was conducted in the absence of any commercial or financial relationships that could be construed as a potential conflict of interest.

## Abbreviations

CF: cystic fibrosis
TSB: Trypticase soy broth
MHA: Mueller-Hinton agar
PFGE: pulsed-field gel electrophoresis
MGT: mean generation time
SBF: specific biofilm formation index
MOI: multiplicity of infection.
